# Circulating Tumor Cell Is a Clinical Indicator of Pretransplant Radiofrequency Ablation for Patients with Hepatocellular Carcinoma

**DOI:** 10.1155/2021/7776389

**Published:** 2021-10-19

**Authors:** Zhitao Chen, Tielong Wang, Chuanbao Chen, Xitao Hong, Jia Yu, Yihao Ma, Yiwen Guo, Changjun Huang, Xiaoshun He, Weiqiang Ju, Maogen Chen

**Affiliations:** Organ Transplant Center, First Affiliated Hospital of Sun Yat-sen University, Guangdong Provincial Key Laboratory of Organ Donation and Transplant Immunology, Guangdong Provincial International Cooperation Base of Science and Technology (Organ Transplantation), Guangzhou 510080, China

## Abstract

**Introduction:**

It is of great significance to confirm reliable indicators for the guidance of pretransplant radiofrequency ablation (RFA) for hepatocellular carcinoma (HCC). In this study, we aim to investigate whether circulating tumor cell (CTC) status is a clinical indicator for RFA before liver transplantation (LT) in HCC patients.

**Method:**

CTC analyses were measured in 79 HCC patients. Clinical outcomes including progression-free (PFS) and overall survival (OS) were compared and analyzed between patients with and without pretransplant RFA.

**Result:**

Forty-two patients were detected as CTC-positive and 18 patients received pretransplant RFA. Recurrence was correlated with CTC count (*P*=0.024), tumor number (*P*=0.035), liver cirrhosis (*P*=0.001), Milan criteria (*P*=0.003), and University of California San Francisco (UCSF) criteria (*P*=0.001). Kaplan–Meier analysis revealed that patients with CTC-positive had a lower PFS rate (*P*=0.0257). For CTC-positive patients, the PFS rate of the pretransplant RFA group was significantly higher than the non-pretransplant RFA group (100% vs. 46.7%, *P*=0.0236). For CTC-negative patients, both PFS rate and OS rate were similar and without significant differences. In multivariate analysis, pretransplant RFA was the independent factor for PFS (*P*=0.025).

**Conclusion:**

Pretransplant CTC status can guide the administration of pretransplant RFA in HCC patients which can improve PFS in CTC-positive HCC patients.

## 1. Introduction

Hepatocellular carcinoma (HCC) is regarded as the most common malignancy and is a leading cause of cancer-related death in the world as the 6^th^ most common worldwide and the 4^th^ leading cancer-related death [1, 2]. In China, HCC is the fourth most diagnosed cancer and the fourth leading cause of cancer death [3]. Treatment of HCC should be carefully selected to achieve promising outcomes. Hepatic resection (HR) is considered as the first-line treatment for patients without vasculature invasion in China [4, 5]. However, the numbers of patients who are suitable for radical resection are limited and the overall 5-year recurrence rate remains high [6]. Liver transplantation (LT) has been accepted as the most effective and curative treatment for patients with both HCC and decompensated cirrhosis [7]. In patients for whom transplantation is not an option (tumor size and numbers is beyond Milan criteria), local and systemic treatment are available as bridging therapy for HCC. Thermal ablation, for example, radiofrequency ablation (RFA), is considered as the preferred treatment for local tumor control and used for bridging or downstaging HCC patients before LT [8]. However, the current clinical use of RFA depends on the experiences based on the traditional tumor characteristics, like tumor size, tumor numbers, and alpha-fetoprotein (AFP), and whether patients will benefit from pretransplant RFA remains controversial [9–12]. Therefore, it is of great significance to confirm reliable indicators for the guidance of pretransplant RFA for HCC.

Our previous study confirmed that positive circulating tumor cell (CTC) count (>1/3.2 ml whole blood) was related to the early recurrence of patients with HCC after LT and showed that pretransplant CTC status may be useful to predict recurrence [13]. Whether it also is useful to guide the application of pretransplant RFA remains unclear. In this study, we aim to investigate whether CTC status is a clinical indicator for RFA before LT in patients with HCC.

## 2. Materials and Methods

### 2.1. Patient Enrollment

**Figure 1:**
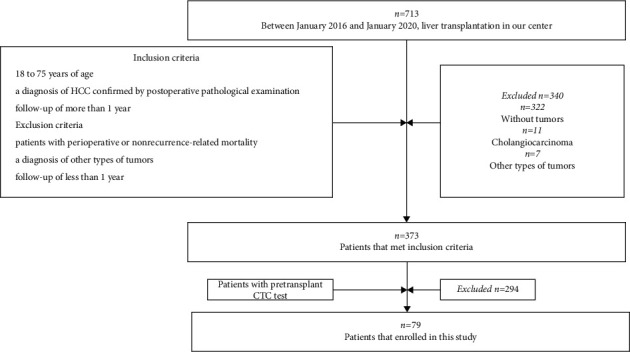
Flowchart for patients' selection in this study.

Between January 2016 and January 2020, 713 patients received LT in our center and 373 patients met inclusion criteria. The inclusion criteria were as follows: 18 to 75 years of age, a diagnosis of HCC confirmed by postoperative pathological examination, and follow-up of more than 1 year. The exclusion criteria were as follows: patients with perioperative or nonrecurrence-related mortality, a diagnosis of other types of tumors, and follow-up of less than 1 year (Figure 1). Afterward, 79 of 373 patients who were tested for CTCs were enrolled in this study. Eighteen patients received only RFA 6 months before LT.

All the procedures were performed in accordance with the ethical standards of the responsible committee on human experimentation (institutional and national) and the Helsinki Declaration of 1964 and later versions. The study was approved by the Institutional Ethics Committee for Clinical Research and Animal Trials of the First Affiliated Hospital of Sun Yat-sen University and an informed consent waiver was granted by the IEC given the retrospective, minimal risk nature of the study. No organs from executed prisoners were transplanted into any of the patients reported in this study.

### 2.2. Perioperative Management and Follow-Up

The immunosuppressive regimen after LT was tacrolimus (Tac) + mycophenolate mofetil (MMF). The followed-up period was at least 1 year. Postoperative visits were performed on postoperative days (POD) 1–7, POD 14, and each postoperative month (POM). Laboratory tests, imaging examinations, and tumor markers were documented. Routine Doppler ultrasound of the liver graft blood flow and the biliary tract was performed once every 2 days for 7 days. Afterward, imaging studies were performed based on patients' clinical status or laboratory findings. HCC recurrence was diagnosed according to the Guidelines for the Diagnosis and Treatment of Primary Liver Cancer (2019 edition) in China [14]. For deceased patients/patients with recurrences, the date of death/recurrence was used as the last follow-up for overall survival (OS) and progression-free survival (PFS), respectively. The follow-up deadline was January 1, 2021.

### 2.3. CTCs Detection

The specific method has been described in the previous study [13]. In brief, the samples (3.2 ml peripheral whole blood collected from a median cubital vein) for CTCs analysis were collected within 1 month before LT. Negative enrichment and imFISH methods were introduced to detect CTCs. The identification of enriched CTCs was performed by imFISH, which combined the FISH probes with chromosome 8 (orange) centromere probes (Abbott Molecular Diagnostics, Des Plaines, IL, USA) and anti-CD45 monoclonal antibodies (Red, Cyttel). To be considered positive, CTCs needed to be hyperdiploid and have the phenotype CEP8+/DAPI+/CD45−. The cutoff value of the CTC count was 1. It was defined as positive as the CTC count was ≥1.

### 2.4. RFA Procedures

Indications for pretransplant RFA were primarily evaluated by physicians [15]. To eliminate selection bias, CTC result was not taken into consideration. Briefly, artificial ascites was created firstly by injecting with 100 ml 5% glucose solution to separate gastrointestinal tract and liver. An 18 G biopsy needle was used for biopsy of lesion sent for pathological examination. Afterward, the lesions were ablated with anhydrous alcohol and injected with 3 ml anhydrous alcohol by 21G PTC needle. RFA was then performed with Cool-tip^TM^ electrode needle (ACT2020) for 10–30 minutes. The primary endpoint of RFA is to obtain a complete necrosis of liver tumors and create a safety margin of at least 10 mm round the external margin of the lesion. Contrast enhanced ultrasound (CEUS) was performed on the second day after ablation to confirm the margin of tumor necrosis.

### 2.5. Statistical Analysis

All statistical analyses of the data were performed by SPSS version 26.0. All data are expressed as the number and percentage of patients. For comparison between groups, the chi-square and Fisher's exact tests were performed for frequencies and continuous data, respectively. Cox proportional hazards model was performed for multivariate analysis. Overall and disease-free survival were compared using the Kaplan–Meier method. A *P* value <0.05 was considered statistically significant.

## 3. Results

### 3.1. Baseline Characteristics of Patients with HCC in CTC-Test Group

To eliminate selection bias, we compared baseline data between the CTC-test group and no CTC-test group (Supplementary Table 1) and there were no significant differences in age, gender, AFP, diagnosis with cirrhosis, and TNM staging between groups (*P* > 0.05). Baseline characteristics of 79 patients enrolled in this study are presented in Table 1. The median follow-up time was 15.7 and 17.3 months for PFS and OS, respectively. Of the 79 patients, 42 patients (53.2%) were detected as CTC-positive (>1/3.2 ml whole blood) and 18 (22.8%) patients received pretransplant RFA only. Besides that, 28 (35.4%) patients did not receive any pre-LT treatment, 3 (3.7%) patients received hepatic resection only, 20 (25.3%) patients received transcatheter arterial chemoembolization (TACE) only, and 10 patients (12.6%) received combined pre-LT treatments. Fifteen patients (19.0%) had multinodular tumors and 31 (39.2%) patients had tumors of larger size (>3 cm). Most patients were diagnosed with liver cirrhosis (92.4%) and hepatic B virus (HBV) infection (82.3%).

### 3.2. CTC Result Is Related to the Early Recurrence of Patients with HCC after LT

**Figure 2:**
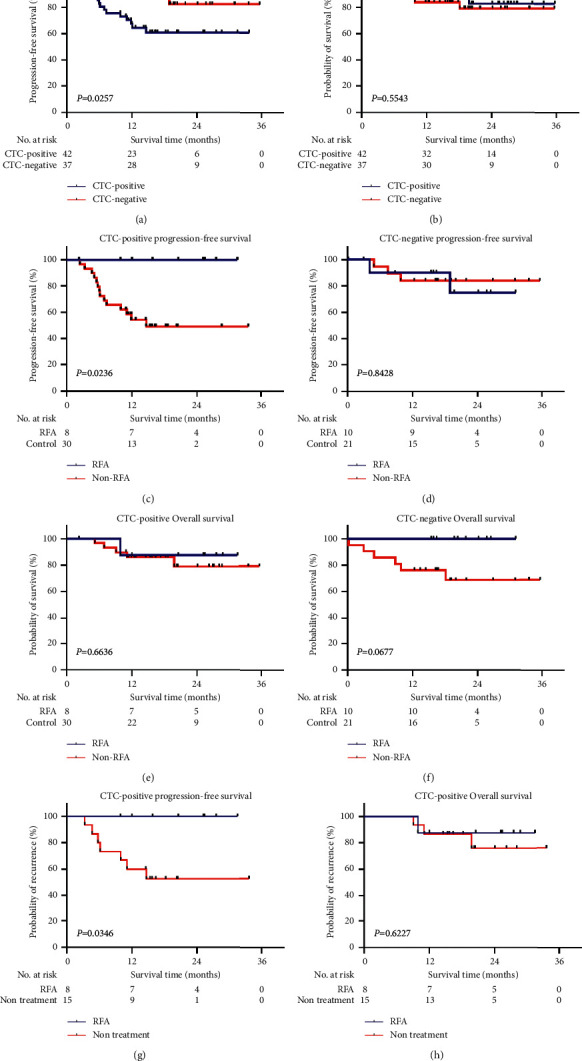
Comparison of PFS and OS between different groups of HCC patients. (a) PFS between CTC-positive and CTC-negative groups; (b) OS between CTC-positive and CTC-negative groups; (c) PFS between RFA and non-RFA groups in CTC-positive HCC patients; (d) PFS between RFA and non-RFA groups in CTC- negative HCC patients; (e) OS between RFA and non-RFA groups in CTC-positive HCC patients; (f) OS between RFA and non-RFA groups in CTC-negative HCC patients. (g) PFS between RFA and nontreatment groups in CTC-positive HCC patients; (h) OS between RFA and nontreatment groups in CTC-positive HCC patients.

Analysis of the 79 patients revealed that 20 (25.3%) patients had a recurrence after LT (Table 2). Fifteen of 20 (75%) patients with recurrence and 27 of 59 (45.7%) patients without recurrence were positive for CTCs, respectively. The results showed that recurrence was correlated with CTC count (*χ*^2^=5.128*P*=0.024), tumor number (*χ*^2^=4.464, *P*=0.035), liver cirrhosis (*χ*^2^=11.559, *P*=0.001), Milan criteria (*χ*^2^=8.773, *P*=0.003), and University of California San Francisco (UCSF) criteria (*χ*^2^=10.225, *P*=0.001), while there were no significant differences in other groups like preoperative AFP (*χ*^2^=1.328, *P*=0.249). The Kaplan–Meier analysis revealed that CTC-positive patients had a lower PFS rate compared with CTC-negative patients (*P*=0.0257; Figure 2(a)). However, the OS rate seemed to be similar and not significantly different between CTC-negative and CTC-positive groups (*P*=0.5543, Figure 2(b)).

### 3.3. Pretransplant RFA Improves PFS in CTC-Positive Patients

Baseline characteristics in HCC patients with or without RFA are shown in Table 3 and no significant differences were found between groups. The association between pretransplant RFA and posttransplant tumor recurrence was analyzed in HCC patients stratified be CTC status. During the follow-up period, recurrence was observed in 15 of 42 CTC-positive patients and 5 of 37 CTC-negative patients, respectively. For CTC-positive patients, the PFS rate of pretransplant RFA group were significantly higher than non-RFA group (100% vs. 46.7%, *P*=0.0236; Figure 2(c)), whereas the OS rates between the groups were similar (87.5% vs. 83.3%, *P*=0.5543; Figure 2(d)). For CTC-negative patients, both PFS rate and OS rate were similar and without significant differences (*P*=0.6636 and 0.0677, respectively; Figures 2(e) and 2(f)). We also had the comparison between RFA group and nontreatment group in CTC-positive patients. The PFS rate of pretransplant RFA group was significantly higher than nontreatment group (nontreatment means LT directly after diagnosis without other preoperative treatment, 100% vs. 46.7%, *P*=0.0346; Figure 2(g)), and the OS rates between the groups were similar (87.5% vs. 80%, *P*=0.6277; Figure 2(h)).

**Figure 3:**
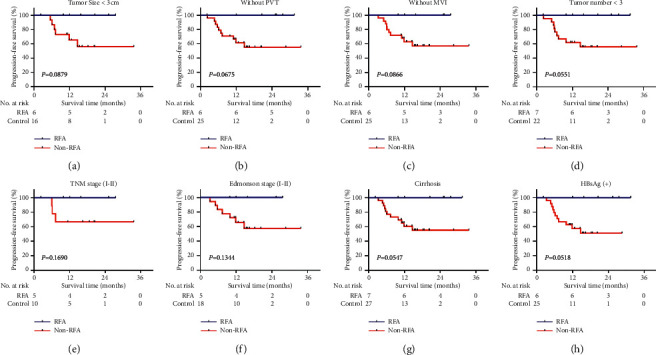
Kaplan–Meier analysis of PFS in subgroups of CTC-positive HCC patients. (a) Tumor size <3 cm. (b) Without PVT. (c) Without MVI. (d) Tumor number <3. (e) Tumor stage (I-II). (f) Edmonson stage (I-II). (g) With cirrhosis. (h) HBsAg (+). PVT: portal vein thrombosis; MVI: microvascular invasions; HBsAg: hepatitis B surface antigen.

Furthermore, the predictive value of CTC-positive for benefit of pretransplant RFA was evaluated within clinical subgroups (Figure 3). The result showed that the PFS rates were higher in patients with pretransplant RFA. However, no significant differences were found between patients with/without pretransplant RFA in these subgroups. The Kaplan–Meier survival analyses for clinical subgroups are shown in Figures 3(a)–3(h). The efficacy of RFA to PFS and OS in CTC-positive HCC patients were also evaluated in multivariate analysis. The result showed that pretransplant RFA was the independent factor for PFS but not for OS (*P*=0.025 and 0.382, respectively; Table 4).

## 4. Discussion

LT has been regarded as the only curative method for patients with HCC. However, posttransplant tumor recurrence was the major limitation for the survival of these patients [16, 17]. RFA is widely used for bridging or downstaging HCC patients before LT [18]. Whether patients will benefit from pretransplant RFA for the lack of reliable indicators remains controversial [19–21]. In this retrospective study, we aimed to investigate whether the CTC result could indicate the application of pretransplant RFA for HCC patients. Overall, our result showed that pretransplant RFA reduces recurrence effectively in CTC-positive patients with HCC. However, For CTC-negative patients, pretransplant RFA cannot reduce both PFS and OS rates. Therefore, the pretransplant CTC result may be used as an indicator for RFA before LT for patients with HCC.

Recurrences are the most negative factor affecting survival for LT patients with HCC [7, 22]. The main cause of recurrence is tumor cell dissemination via blood vessel infiltration [23]. In our study, recurrence after LT was related to CTC count, Milan criteria, and UCSF criteria. The recurrence of LT within the Milan criteria was 13.0%, and it is better than 43.6% for those beyond the Milan criteria. The recurrence of LT within the UCSF criteria was 13.7% and it is also better than 46.4% for those beyond UCSF criteria. This indicates that Milan criteria and UCSF criteria are promising criteria for favorable outcomes [24–26]. However, in China, patients tend to have HR or conservative treatment due to economic or ideological reasons, even if tumors are detected early. LT would be considered only when other treatments were ineffective or if the tumor progressed. Therefore, finding a method to predict the prognosis after LT is of great significance. Imaging, pathological examinations, and common serum markers like AFP have their limitations in diagnostic accuracy and sensitivity and a novel diagnostic method is needed [27]. CTCs were first discovered and described by Ashworth et al. in 1869 [28]. Vona et al. first explored the prognostic value of blood CTCs in patients with HCC and it was related to prognosis and recurrence in patients with HCC [29]. CTC detection can be applied as a method for early cancer detection and prediction of recurrence or metastasis risk [30–32]. Compared with the conventional clinicopathological index like AFP, CTC has the advantage of predicting microvascular invasion and dynamical detection [33]. Castro-Giner and Aceto showed in their study that it can serve as a promising tool to provide insights into the biology of metastatic cancers and with potential for use in liquid biopsy-based personalized cancer treatment [34]. Ramirez et al. showed in their study that CTC was an essential key for the management of the patients with HCC on the waiting list for liver transplantation [35]. Zhou et al. found CTCs can indicate the prognosis of HCC for its efficacy in predicting microvascular invasion [36]. Our previous study also revealed that CTC-positive patients had a worse prognosis after LT than CTC-negative patients [13]. In the current study, it remains unclear whether patients can benefit from RFA by achieving a high degree of tumor necrosis before LT [37]. Agopian et al. showed in their study including 3601 recipients of LT that none of the significant differences were identified in survival between patients with/without locoregional treatment (LRT) prior to LT [8]. However, in his study, only 10% of patients received ablation therapy and only 10% of patients had HCC secondary to HBV. Different types of HCC have different responses to the preoperative treatment. A small percentage of pretransplant RFA and HBV related HCC might be the reason for its failure to get comparable results. Our results showed that pretransplant RFA improves PFS effectively in CTC-positive patients with HCC. However, in clinical subgroups, significant differences were not found between patients with/without pretransplant RFA. We consider these results in traditional clinical subgroups were not in conflict with previous studies [38, 39], for CTC result is a novel biomarker to evaluate the risk of recurrence and may be a complementary biomarker of traditional clinical indicators to pretransplant RFA in HCC patients. It can be applied to the guidance for the downstaging treatment before LT [40]. In addition, the CTC result can be used to guide the pretransplant management for HCC patients. In our study, CTCs were detected in 42 of 79 (53.2%) patients before LT. The sensitivity and specificity of CTCs detection were 75% and 54.2%, respectively. The results showed that the CTCs test had good sensitivity and specificity so it could be helpful to predict recurrence. An additional pretransplant RFA may not be necessarily needed for CTC-negative patients. A further prospective, multicenter, and large population study is needed to investigate the value of the CTC result as an indicator in guiding pretransplant treatment.

RFA was first applied and described by Rossi et al. in 1993 [41]. In China, the use of RFA for HCC was quickly developed in recent years. Compared with HR, RFA is minimally invasive and has lower morbidity and mortality rates, especially in cases with impaired liver functions. However, the tumor size and stage are important factors for the outcome of RFA [42, 43]. Yan et al. showed in their study that a larger tumor size (>5 cm) would result in a less complete necrosis rate and RFA alone for HCC is limited [44]. The combination of RFA and other methods would have further benefits for patients. RFA can be used for bridging or downstaging HCC patients before LT; therefore, it may help to prolong time on the waiting list and reduce the waiting list mortality rate [45]. The current clinical use of RFA depends on the experiences based on the traditional tumor characteristics and a novel biomarker to guide pretransplant treatment is of great significance. Our result showed that transplant RFA improves PFS effectively of patients in the status of CTC-positive. For CTC-negative patients, pretransplant RFA did not improve the early PFS rate, and this suggested that RFA may not be necessary for such patients. For the overall survival comparison, it seems like the CTC-negative patients with pretransplant RFA got a better OS than non-RFA counterparts (100% vs. 76.2%), however without significant difference (*P*=0.0677). Small sample size and short follow-up period may be the reason. Enlarging sample scale and prolonging follow-up period are needed for convincing result. Furthermore, we made a multivariate analysis and figured out that pretransplant RFA was the independent factor for PFS.

Our study has limitations. First, the sample size is small and from a single-center institution. Larger multicenter studies are needed to determine whether pretransplant RFA can improve PFS in patients with HCC. Second, the value of postoperative CTCs in guiding pretransplant RFA should be analyzed in further study. For future studies, the 3-year and 5-year PFS and OS values should be calculated to obtain more convincing conclusions.

## 5. Conclusion

In conclusion, this study provides evidence that CTC results can be used for guiding pretransplant RFA for patients with HCC. Therefore, the CTC result is a potentially promising biomarker and clinical indicator for the administration of pretransplant RFA for HCC patients.

## Figures and Tables

**Table 1:** Baseline characteristics of HCC patients for the entire study.

Variable		*N* = 79	
		*n*	%
Gender	Male	74	93.7
	Female	5	6.3

Age (years)	>50	47	59.5
	≤50	32	40.5

CTC count	>1	42	53.2
	≤1	37	46.8

Tumor number	>3	15	19.0
	≤3	64	81.0

Tumor diameter (cm)	>3	31	39.2
	≤3	48	60.8

PVT	Yes	16	20.3
	No	63	79.7

MVI	Yes	15	19.0
	No	64	81.0

Edmonson stage	I-II	48	60.8
	III-IV	31	39.2

Liver cirrhosis	Yes	73	92.4
	No	6	7.6

Milan criteria	Yes	46	58.2
	No	33	41.7

UCSF criteria	Yes	51	64.6
	No	28	35.4

HBsAg (+)	Yes	65	82.3
	No	14	17.7

AFP (ng/ml)	>400	20	25.3
	≤400	59	74.7

TNM stage	I	10	12.7
	II	23	29.1
	III-IV	46	58.2

Pretransplantation treatment	Yes	51	64.6
	No	28	35.4

RFA only	Yes	18	22.8
	No	61	77.2

Recurrence	Yes	20	25.3
	No	59	74.7

AFP: alpha-fetoprotein; CTC: circulating tumor cells; HBsAg: hepatitis B surface antigen; HCC: hepatocellular carcinoma; MVI: microvascular invasions; PVT: portal vein thrombosis; RFA: radiofrequency ablation; UCSF: University of California San Francisco.

**Table 2:** Analysis of relevant factors for recurrence of HCC in 79 patients.

Variable		*N* = 79			
		Recurrence (*n* = 20)	Nonrecurrence (*n* = 59)	*χ* ^2^	*P* value
Gender, *n* (%)	Male	19 (24.1)	55 (69.6)	0.08	0.778
	Female	1 (1.3)	4 (5.1)		

Age (years), *n* (%)	>50	11 (13.9)	36 (45.6)	0.224	0.636
	≤50	9 (11.4)	23 (29.1)		

CTC count, *n* (%)	>1	15 (19.0)	27 (30.4)	5.128	**0.024**
	≤1	5 (6.3)	32 (40.5)		

Tumor number, *n* (%)	>3	7 (8.9)	8 (10.1)	4.464	**0.035**
	≤3	13 (16.5)	51 (64.6)		

Tumor diameter (cm), *n* (%)	>3	10 (12.7)	19 (24.1)	2.036	0.154
	≤3	10 (12.7)	40 (50.6)		

PVT, *n* (%)	Yes	6 (7.6)	10 (12.7)	1.575	0.209
	No	14 (17.7)	49 (62.0)		

MVI, *n* (%)	Yes	7 (8.9)	10 (12.7)	2.882	0.090
	No	13 (16.5)	49 (62.0)		

Edmonson stage, *n* (%)	I-II	9 (11.4)	39 (49.4)	2.790	0.095
	III-IV	11 (13.9)	20 (25.3)		

Liver cirrhosis, *n* (%)	Yes	15 (19.0)	58 (73.4)	11.559	**0.001**
	No	5 (6.3)	1 (1.3)		

Milan criteria, *n* (%)	Yes	6 (7.6)	40 (50.6)	8.773	**0.003**
	No	14 (17.7)	19 (24.0)		

UCSF criteria, *n* (%)	Yes	7 (8.8)	44 (55.6)	10.225	**0.001**
	No	13 (16.4)	15 (18.9)		

HBsAg (+), *n* (%)	Yes	16 (20.3)	49 (62.0)	0.095	0.757
	No	4 (5.1)	10 (12.7)		

RFA only, *n* (%)	Yes	2 (2.5)	16 (20.3)	2.448	0.115
	No	18 (22.8)	43 (54.4)		

AFP (ng/ml), *n* (%)	>400	7 (8.9)	13 (16.5)	1.328	0.249
	≤400	13 (16.5)	46 (58.2)		

TNM stage, *n* (%)	I	0	10 (12.7)	6.334	**0.042**
	II	4 (5.1)	19 (24.1)		
	III-IV	16 (20.3)	30 (38.0)		

Bold *P* values indicate statistical significance. AFP: alpha-fetoprotein; CTC: circulating tumor cells; HBsAg: hepatitis B surface antigen; HCC: hepatocellular carcinoma; MVI: microvascular invasions; PVT: portal vein thrombosis; RFA: radiofrequency ablation; UCSF: University of California San Francisco.

**Table 3:** Baseline characteristics in HCC patients with/without RFA.

Variable		*N* = 79			
		RFA (*n* = 18)	Non-RFA (*n* = 61)	*χ* ^2^	*P* value
Gender, *n* (%)	Male	16 (20.3)	58 (73.4)	0.899	0.343
	Female	2 (2.5)	3 (3.8)		

Age (years), *n* (%)	>50	8 (10.1)	39 (49.4)	2.191	0.139
	≤50	10 (12.7)	22 (27.8)		

CTC count, *n* (%)	>1	8 (10.1)	34 (43.0)	0.712	0.399
	≤1	10 (12.7)	27 (34.2)		

Tumor number, *n* (%)	>3	1 (1.3)	14 (17.7)	2.734	0.098
	≤3	17 (21.5)	47 (59.5)		

Tumor diameter (cm), *n* (%)	>3	6 (7.6)	9 (11.4)	3.119	0.077
	≤3	12 (15.2)	52 (65.8)		

PVT, *n* (%)	Yes	3 (3.8)	13 (16.5)	0.186	0.667
	No	15 (19.0)	48 (60.8)		

MVI, *n* (%)	Yes	3 (3.8)	12 (15.2)	0.082	0.775
	No	15 (19.0)	49 (62.0)		

Edmonson stage, *n* (%)	I-II	11 (13.9)	37 (46.8)	0.001	0.972
	III-IV	7 (8.9)	24 (30.4)		

Liver cirrhosis, *n* (%)	Yes	17 (21.5)	56 (71.0)	0.138	0.710
	No	1 (1.3)	5 (6.3)		

Milan criteria, *n* (%)	Yes	14 (11.4)	32 (40.5)	3.663	0.056
	No	4 (5.0)	29 (36.7)		

UCSF criteria, *n* (%)	Yes	15 (18.9)	36 (45.5)	3.592	0.058
	No	3 (3.7)	25 (31.6)		

HBsAg (+), *n* (%)	Yes	14 (17.7)	51 (64.6)	0.324	0.569
	No	4 (5.1)	10 (12.7)		

AFP (ng/ml), *n* (%)	>400	3 (3.8)	17 (21.5)	0.922	0.337
	≤400	15 (19.0)	44 (55.7)		

TNM stage, *n* (%)	I	1 (1.3)	9 (11.4)	1.723	0.423
	II	7 (8.9)	16 (20.3)		
	III-IV	10 (12.7)	36 (45.6)		

AFP: alpha-fetoprotein; CTC: circulating tumor cells; HBsAg: hepatitis B surface antigen; HCC: hepatocellular carcinoma; MVI: microvascular invasions; PVT: portal vein thrombosis; RFA: radiofrequency ablation; UCSF: University of California San Francisco.

**Table 4:** Multivariate analysis to identify independent risk factors of progression-free survival and overall survival in CTC-positive HCC patients.

Variable	Progression-free survival		Overall survival	
	HR (95% CI)	*P* value	HR (95% CI)	*P* value
Tumor number (>3)	3.059 (0.672∼13.394)	0.148	0.380 (0.005∼30.134)	0.664
Tumor size (>3 cm)	0.348 (0.051∼2.395)	0.284	2098.606 (0∼5.316^*∗*^10^^9^)	0.934
Edmonson stage (III-IV)	0.693 (0.136∼3.523)	0.659	1.297 (0.068∼24.657)	0.863
TNM stage (III-IV)	1.394 (0.340∼5.721)	0.644	8.755 (0.272∼281.503)	0.220
AFP (>400 ng/ml)	2.047 (0.340∼12.329)	0.434	1.944 (0.04∼91.723)	0.735
HBsAg (+)	0.348 (0.054∼2.259)	0.269	0.045 (0.000∼61.85)	0.216
Pretransplant RFA (yes)	0.076 (0.008∼0.724)	**0.025**	0.140 (0.002∼11.449)	0.382

Bold *P* values indicate statistical significance. AFP: alpha-fetoprotein; CTC: circulating tumor cells; HBsAg: hepatitis B surface antigen; HCC: hepatocellular carcinoma; MVI: microvascular invasions; PVT: portal vein thrombosis; RFA: radiofrequency ablation; UCSF: University of California San Francisco.

## Data Availability

The data that support the findings of this study are available from the corresponding author upon reasonable request.

## Abbreviations

AFP: Alpha-fetoprotein
CTC: Circulating tumor cell
HCC: Hepatocellular carcinoma
HR: Hepatic resection
LRT: Locoregional treatment
MMF: Mycophenolate mofetil
OS: Overall survival
PFS: Progression-free survival
POD: Postoperative day
RFA: Radiofrequency ablation
Tac: Tacrolimus
UCSF: University of California San Francisco.

## Supplementary Materials

Supplementary Materials Table 1: baseline data comparison between no CTC-test and CTC-test patients with HCC.
