# Supplementary material for: Circulating Tumor Cell Is a Clinical Indicator of Pretransplant Radiofrequency Ablation for Patients with Hepatocellular Carcinoma
Source: J Oncol. 2021 Oct 19;2021:7776389. doi: 10.1155/2021/7776389 (PMC8548160; doi:10.1155/2021/7776389)
Supplement: Supplementary Materials — Table 1: baseline data comparison between no CTC-test and CTC-test patients with HCC. [file 7776389.f1.docx]

**Table 1 Baseline data comparison between No CTC-test and CTC- test patients with HCC**

| Variables | CTC-test  (n=79) | No CTC-test  (n=294) | ꭓ^2^ | *P*-value |
| --- | --- | --- | --- | --- |
| Age，≥50 ys, n(%) | 47(12.6) | 158(42.4) | 0.832 | 0.362 |
| Gender, male, n (%) | 74(19.8) | 266(71.3) | 0.788 | 0.375 |
| AFP＞400ug/L, n (%) | 20(5.4) | 94(25.2) | 1.300 | 0.254 |
| Cirrhosis, n (%) | 73(19.6) | 268(71.8) | 0.124 | 0.725 |
| TNM staging, n (%) |  |  | 0.836 | 0.658 |
| I | 10(2.7) | 30(8.0) |  |  |
| II | 23(6.2) | 77(20.6) |  |  |
| III-IV | 46(12.3) | 187(50.1) |  |  |

Abbreviations: AFP, alpha-fetoprotein; CTC, circulating tumor cells; HCC, hepatocellular carcinoma.
